# Multi-Omics Technologies Applied to Improve Tick Research

**DOI:** 10.3390/microorganisms13040795

**Published:** 2025-03-31

**Authors:** Arlex Rodríguez-Durán, Vinícius Andrade-Silva, Muhammad Numan, Jéssica Waldman, Abid Ali, Carlos Logullo, Itabajara da Silva Vaz Junior, Luís Fernando Parizi

**Affiliations:** 1 Programa de Pós-Graduação em Ciências Veterinária, Universidade Federal do Rio Grande do Sul, Avenida Bento Gonçalves, 9090, Porto Alegre 91540-000, RS, Brazil; arrodriguezdu@unal.edu.co (A.R.-D.); mn.awkum@gmail.com (M.N.); 2 Centro de Biotecnologia, Universidade Federal do Rio Grande do Sul, Avenida Bento Gonçalves, 9500, Porto Alegre 91501-970, RS, Brazil; vinicius21126@gmail.com (V.A.-S.); jessica.waldman@hotmail.com (J.W.); itabajara.vaz@ufrgs.br (I.d.S.V.J.); 3 Grupo de Investigación Parasitología Veterinaria, Laboratorio de Parasitología Veterinaria, Universidad Nacional de Colombia (UNAL), Carrera 30 No 45-03, Bogotá 110111, Colombia; 4 Department of Zoology, Abdul Wali Khan University Mardan, Mardan 23200, Khyber Pakhtunkhwa, Pakistan; uop_ali@yahoo.com; 5 Instituto de Bioquímica Médica Leopoldo de Meis, Universidade Federal do Rio de Janeiro, Rio de Janeiro 21941-902, RJ, Brazil; carloslogullo@yahoo.com.br; 6 Instituto Nacional de Ciência e Tecnologia em Entomologia Molecular (INCT-EM), Rio de Janeiro 21941-853, RJ, Brazil; 7 Faculdade de Veterinária, Universidade Federal do Rio Grande do Sul, Avenida Bento Gonçalves, 9090, Porto Alegre 91540-000, RS, Brazil

**Keywords:** Argasidae, biology, control, Ixodidae, microbiota, physiology, vaccine

## Abstract

The advancement of multi-omics technologies is crucial to deepen knowledge on tick biology. These approaches, used to study diverse phenomena, are applied to experiments that aim to understand changes in gene transcription, protein function, cellular processes, and prediction of systems at global biological levels. This review addressed the application of omics data to investigate and elucidate tick physiological processes, such as feeding, digestion, reproduction, neuronal, endocrine systems, understanding population dynamics, transmitted pathogens, control, and identifying new vaccine targets. Furthermore, new therapeutic perspectives using tick bioactive molecules, such as anti-inflammatory, analgesic, and antitumor, were summarized. Taken together, the application of omics technologies can help to understand the protein functions and biological behavior of ticks, as well as the identification of potential new antigens influencing the development of alternative control strategies and, consequently, the tick-borne disease prevention in veterinary and public health contexts. Finally, tick population dynamics have been determined through a combination of environmental factors, host availability, and genetic adaptations, and recent advances in omics technologies have improved our understanding of their ecological resilience and resistance mechanisms. Future directions point to the integration of spatial omics and artificial intelligence to further unravel tick biology and improve control strategies.

## 1. Introduction

The mapping and sequencing of the human genome stimulated the development of new technologies for DNA sequencing, improving the characterization and identification of the composition and function of gene products of various living organisms [1]. In this sense, molecular tools were developed and/or improved to expand knowledge of proteomics, transcriptomics, genomics, metabolomics, lipidomics, and epigenomics, which correspond to global analysis of proteins, RNA, genes, metabolites, lipids, and methylated DNA or modified histone proteins in the chromosomes and are designated by the suffix “omics” [2,3].

In recent years, new tools for molecular analysis have been developed that allow for a comprehensive characterization of the spatial organization of molecules, cells within tissues, or transcriptomes of a set of cells, which has allowed for a better understanding of the biology of different organisms [4]. An example of the application of these technologies has been in the study of ticks, which are the first vector of pathogens in animals and the second vector in humans [5]. This has facilitated the sequencing and assembly of genomes of different tick species around the world [6,7].

These tools have improved the quality of tick genomic analyses, allowing for progress in the biological science of these arthropods. These high-quality genomes (at chromosome-level genome assembly) have allowed for the elucidation of new epigenetic functions, the expansion of gene families, and the deciphering of genetic variations between tick species. In addition, the relationship with their microbiota and the pathogens they transmit, among other findings, allows for a deeper understanding of these vectors [8,9,10]. Therefore, this review summarizes and discusses multi-omics information that can provide deeper insight into the coordination of various and intricate host–tick–pathogen interactions, tick population spread dynamics, and the development of new therapeutic drugs.

## 2. Methods

### 2.1. Information Search Strategy

The previously proposed methodologies by Moher et al. [11], Aromataris et al. [12], and Page et al. [13] were used to collect the information used in this review. Data were extracted from the PudMed, Scopus, SciELO, ScienceDirect, and Redalyc databases using the search terms “omics” and “tick”. The search was conducted between May 2024 and January 2025.

### 2.2. Inclusion and Exclusion Criteria

Studies providing omics data to investigate and elucidate the physiological and biological processes of ticks, such as feeding, digestion, reproduction, and neural and endocrine systems, population dynamics, transmitted pathogens, control strategies, and identification of new vaccine targets were included. In addition, studies published in English, Portuguese, or Spanish were selected. Studies not published in indexed journals, books, or non-peer-reviewed sources were not included in the review. The publication year of the studies was not restricted, as the use of omics tools in tick research spans less than 30 years.

### 2.3. Number of Studies

Based on the search strategy and the proposed criteria, a total of 541 results were identified, of which 244 were eligible for evaluation. Of these, information from 181 studies stored across the five databases was used (see the overlap rate between sources in Figure S1).

## 3. Recent Omics Studies That Improved Knowledge About Tick Biology

### 3.1. Embryonic Development

Embryogenesis is the process of embryo development, during which it forms and matures into a larva and, more rarely, a nymph depending on the tick’s species [14]. Transcriptomic studies have allowed for more detailed transcript profiles for each stage of embryonic development in *Rhipicephalus turanicus*, *Haemaphysalis flava*, and *Rhipicephalus microplus* (Table 1) [15,16,17]. For example, for *R. turanicus*, more differentially expressed genes (DEGs) were observed at early embryonic stages compared to later stages, showing stage-specific characteristics [15]. Understanding gene expression profiles could be used in the future to disrupt embryonic development in ticks, which in turn could also affect the transovarial transmission of some pathogens carried by these arthropods during embryogenesis [17].

Likewise, through the use of sequencing techniques, differential transcription profiles during embryogenesis have been identified for *glutathione S-transferases* (*GSTs*) and *ferritins* (*Fers*). Proteins from these genes participate in the detoxification of xenobiotic compounds and oxidative stress, respectively, in ticks [31,32,33]. In *Haemaphysalis longicornis*, high transcription levels of *GST* genes have been identified in eggs, particularly during the first day after oviposition and in the early stage of embryogenesis. In contrast, *Fers* gene transcriptions increase on day 10, reaching a peak on day 15 of embryogenesis [32]. On the other hand, in studies conducted in *Hyalomma rufipes*, high relative transcription of *Fers* in the ovary was found, suggesting that *Fers* may have a more prominent role in ovary function in this tick species [33].

Other functional omics studies using RNA interference (RNAi) techniques have been able to describe glycogen used by *R. microplus* during vitellogenesis and embryogenesis [34,35,36,37]. The results obtained in the characterization of the role of AKT (protein kinase B) and GSK-3 (glycogen synthase kinase-3) in glycogen metabolism and cell viability during embryonic development in the tick species *R. microplus* and *H. longicornis* have shown a conserved role of the AKT/GSK-3 axis in cell survival and glycogen metabolism [37,38]. For example, in *R. microplus*, silencing the *GSK-3* gene using RNAi leads to a reduction in oviposition and hatching of fully engorged female larvae in this tick species [39].

Additionally, the RNAi technique has allowed researchers to describe the function of proteins, such as THAP (Tick Heme-binding Aspartic Protease) [40], phospholipase A_2_ [41], BYC (*Boophilus* yolk cathepsin) [42], poly P (polyphosphate P) [43], NPC1 (Niemann-Pick C1) [44], VTDCE (Vitellin-Degrading Cysteine endopeptidase) [45,46], Bm05br (Brazil *Rhipicephalus microplus* protein 05) [47], PERK (Kinase R-like endoplasmic reticulum) [48], TOR (Rapamycin) [49], PEPCK (Phosphoenolpyruvate carboxykinase enzyme) [50], Salp12 (*Ixodes scapularis* salivary gland protein of 12 kDa) [51], and RmVgR (*Rhipicephalus microplus* vitellogenin receptor) [52]. These genes are associated with functions like vitellogenesis, embryogenesis, transport, metabolism, and important signaling pathways related to reproduction in ticks.

### 3.2. Feeding Process

The increase in tick omics data in recent years has allowed for improving knowledge of the physiological processes of these parasites, especially during feeding (Table 1) [44,53,54,55,56]. These analyses are showing, for example, that several proteins are differentially expressed in the midgut of fed and unfed ticks as enzymes involved in digestion, iron metabolism, and oxidative stress [57,58]. This represents another step towards identifying distinct midgut pathways and metabolic activities, as shown for *A. americanum*, *I. ricinus*, *H. flava,* and *Ornithodoros erraticus* [59,60,61]. Furthermore, the discrimination of different levels of midgut gene transcription during pathogen acquisition, persistence, or transmission is improving the understanding of tick vector competence [62,63,64].

Another organ related to the feeding process is the salivary glands [65,66]. The use of high-throughput RNA sequencing (RNA-seq) for salivary glands allowed for the description of sialomes in an increasing number of tick species today, revealing the abundance and complexity of salivary gland transcriptomes and proteomes [67,68,69]. Omics analyses are very helpful to identify the role of components of saliva, which allows blood uptake through antihemostatic and immunomodulatory activities, in the tick–host interface [70,71,72]. Many protein families involved in the hematophagic processes, such as peptidases or transporter proteins, can be characterized through such an analysis [64]. For example, Jia et al. [64] identified the expansion of the transmembrane protease serine 6 family of matripase 2, which helps counteract oxidative stress; the serine carboxypeptidase, which is involved in nutrient acquisition; and the alcohol dehydrogenase, which plays a role in nutrient metabolism. As a consequence, the differential expression of salivary and midgut proteins during the hematophagic process allows for the adaptation of ticks against different host and/or host defense mechanisms.

Additionally, omics technologies can improve the understanding of diseases caused by tick molecules injected into the hosts during blood feeding. For example, one host allergic reaction developed during *A. americanum* or *I. ricinus* feeding is known as alpha-Gal syndrome (AGS), where IgE antibodies are produced against glycan galactose-alpha-1-3-galactose (alpha-Gal), resulting in skin redness and allergy in more severe cases [73,74]. The exact nature of tick molecules that result in AGS is not fully characterized, but it was proposed that glycolipids with bound alpha-Gal can result in AGS [62]. In this way, by performing a tick salivary proteome and lipidome, it was shown that proteins and lipids lacking alpha-Gal can also be related to AGS [75], improving knowledge about sensitization development.

Finally, new tick saliva proteins that are essential for the feeding and transmission of pathogens are been described by applying methods like liquid chromatography (LC) and mass spectrometry (MS), techniques in the study of proteomics, which have allowed for the analysis of the sialoma of the species *A. americanum*, *Argas monolakensis*, *I. ricinus*, *R. microplus*, and *R. sanguineus* s.l. [18,59,76,77,78,79,80]. Recently, it was observed that the bacterium *Borrelia burgdorferi* (which causes Lyme disease), in order to survive in the species *I. scapularis*, makes modifications to the protein content in the saliva to promote its survival at the tick feeding site [81]. For example, the enzymes copper/zinc superoxide dismutase, which lead to the production of H_2_O_2_, which is toxic to *B. burgdorferi,* were suppressed, while catalase and thioredoxin, which neutralize H_2_O_2_, and pyruvate kinase, which produces pyruvate that protects *B. burgdorferi* from death by H_2_O_2_, were increased [82].

### 3.3. Neural and Endocrine Regulation

G protein-coupled receptors (GPCRs) are transmembrane proteins that mediate signal transduction and biological processes [83]. Omics have provided a wide range of information about the nucleotide and protein or peptide sequences that are necessary for understanding their roles [83]. In humans, GPCRome-wide homology models containing structural and biological activity information are available in a GPCRdb database, allowing for the prediction of six classes of these receptors [84,85,86].

In ticks, GPCRs have already been annotated, and it has been shown that the interaction between these receptors and hormones, neuropeptides, peptide hormones, and lipoglicoproteins, among other ligands, leads to signal transduction, which influences most of the physiological processes [63,83]. A combination of structural-based and alignment-free methods based on sequence similarity allowed for the identification of 112 GPCR candidates in the synganglion of *R. microplus* that were distributed in different families: secretin, glutamate, and rhodopsin [87]. In addition, it has been shown that for every 20 GPCRs, five biogenic amines were identified, a pattern similar across different arthropods. In insects, each neuropeptide can interact with one or two receptors, but, in *I. scapularis*, this number increases 10-fold [63,88], suggesting potential targets for tick control [89].

Previous work has been able to provide identification and characterization of neuropeptide sequences. The presence of these signaling molecules was observed in different hard tick species, with the highest abundance of transcripts encoding neuropeptides being identified in the *R. microplus* synganglion (Table 1) [90]. Corazonin is a conserved neuropeptide involved in arthropod ecdysis [91]. Using a bioinformatic approach, two splice variants of the corazonin receptor were identified in *I. scapularis* [88]. In general, GPCRs do not present an N-terminal signal sequence; however, this signal sequence is present in one of these splice variants, which could aid in the insertion of the receptor into the rough endoplasmic reticulum membrane [88,91].

It must be taken into account that GPCRs as well as their ligands can be duplicated or lose their function throughout evolution [92]. Allatostatin C is a neuropeptide that was first described in *Manduca sexta* and plays a role in inhibiting hormone juvenile synthesis [93,94]. Like the vertebrate neuropeptide somatostatin, allatostatin C acts on GPCR, and both neuropeptide sequences were considered orthologous [94,95]. The alignment of allatostatin sequences from different arthropods showed that these neuropeptides present important structural differences, which allowed them to be grouped into three groups of paralogous peptides: allatostatin C, allatostatin CC, and allatostatin CCC [94,96]. Interestingly, allatostatin C was not identified in the genomic and transcriptomic analysis of tick sequences, suggesting the loss of this peptide during evolution [90]. Thus, understanding new physiological processes that describe the involvement of endocrine regulation, GPCRs, and their ligands may help in the identification of new targets and in the development of alternative tick control strategies.

## 4. Omics Analysis for Tick Bacterial Microbiota

The application of meta-omics through the integration of metatranscriptomics and metaproteomics of some tick species studied worldwide (Table 1) has allowed us to improve our knowledge about the species, abundances, co-occurrences, or associations of the different taxa of bacteria that are part of the microbiota of these arthropods [8,97,98,99]. Advances in next-generation sequencing (NGS) technology have enabled individual analysis of networks that describe the complexity and broader role that bacteria play in tick biology [100,101,102], as well as host/tick–pathogen and host/tick–microbiome interactions [99,101].

Also, by sequencing the microbiome of the 16S rRNA gene in ticks and applying bioinformatics tools, researchers have been able to identify microbial variation in zoonotic pathogens in *I. scapularis* [10], such as *B. burgdorferi* and *Streptococcus* spp. Similarly, the interaction between ticks and their hosts can lead to changes in the tick’s microbiota, as ticks acquire new bacterial genera from their host [103,104]. This fact has been confirmed through RNA sequencing of skin tissues in mice infested with *Dermacentor marginatus* and *Haemaphysalis montgomeryi*, which demonstrated that certain bacterial genera in the skin microbiome of the mice were also found in these tick species [105,106].

Deep sequencing methodologies have played a key role in this accumulation of knowledge, being able to identify and classify different molecules from the tick microbiota. With this information, it has been possible to phylogenetically group bacterial species as endosymbionts, pathogens, clade differentiation, regulation of functions or adaptation, and tick immunity [107,108,109]. For example, bacterial metabolic barcoding targeting the 16S rRNA locus demonstrated that Australian tick species *Amblyomma triguttatum*, *Ixodes antechini*, *Ixodes australiensis*, *Ixodes holocyclus*, *Ixodes tasmani,* and *Ixodes trichosuri* harbor unique and diverse bacterial communities [110,111]. In addition, it reveals taxa of health interest, such as *Anaplasmataceae*, *Bartonella*, *Borrelia*, *Coxiellaceae*, *Francisella*, *Midichloria*, *Mycoplasma,* and *Rickettsia* [112].

By applying metagenomics, it was possible to find out that the microbiome varies between tissues of different tick species. In a study of the microbiome of the midgut and ovaries of the ticks *I. ricinus* and *R. microplus* before, during, and after blood feeding, it was possible to establish that the number of copies of the 16S rDNA of the bacterial species present in the ovarian microbiome of both tick species was higher compared to the copies of 16S rDNA of the midgut microbiome [113,114]. Also, it was possible to demonstrate the instability and deficiency of the midgut microbiome in contrast to the abundant and stable monospecific microbiome of the ovaries in these two tick species [113].

The use of omics technologies allowed us to understand the diversity and variety of the microbiota that exist in the different stages of development of tick species belonging to the Ixodidae and Argasidae families [115,116,117,118,119]. In *Dermacentor silvarum* (Ixodidae), specific bacterial species associated with each stage of development were shown, with the bacterial phylum Actinobacteria being more abundant in nymphs and Proteobacteria in adults [120]. In studies carried out in *Argas persicus* (Argasidae), the bacterial diversity was different, recording the bacterial phylum Actinobacteria in all stages and Proteobacteria only in larvae and nymphs [118].

As omics technology advances, our knowledge of the tick microbiome is improving, as well as our knowledge of how it can affect the acquisition, maintenance, and transmission of pathogens according to different factors, such as the geographic area where the ticks develop [118]. In a study of the microbiome of different populations of the tick species *Dermacentor variabilis* from four regions of the United States (West, Midwest, South, and Northeast), it was found that the geographic location of each tick population consistently influenced bacterial species richness. Specifically, 18 bacterial genera were specific to each region studied [120].

The application of multi-omics strategies in the last decade allowed for developing deeper knowledge of the bacterial microbiota found in ticks by identifying and better understanding the interactions with this arthropod and the pathogens they transmit [99,121,122]. This information could open up new control strategies by generating potential targets for the development of, for example, anti-tick microbiota vaccines [123,124,125,126,127,128], resulting in poor tick fitness through microbiota dysregulation. However, it is still necessary to identify a series of standard marker genes and reference databases that can identify new groups or discover their interaction with this arthropod. Rapidly evolving molecular techniques are expected to help make this understanding a reality in the coming years.

## 5. Therapeutic Advances Through Omics Using Tick Molecules

Utilizing omics technologies, it has been found that specific proteins in ticks could be used as pharmacological candidates in the future [129]. For example, proteomic analysis of salivary glands from partially engorged females of *Haemaphysalis qinghaiensis* allowed for the identification of the protein Hq023 (*Haemaphysalis qinghaiensis* 023). When tested in laboratory mice as an experimental model, this protein demonstrated an analgesic effect [130]. The discovery of this protein could open a new avenue for the development of tick-derived analgesics. Likewise, in the study of the transcriptome of the salivary gland of *Amblyomma cajennense*, using expressed sequence tags (EST), the protein Amblyomin-X (*Amblyomma cajennense* s.l. factor X inhibitory protein) was identified, an inhibitor of the Kunitz-type serine protease, and it was described with cytotoxic functions in various tumor cells [131,132]. A study conducted by Chudzinski-Tavassi et al. [133] on the antitumor activity of Amblyomin-X showed a regression of tumor mass and a decrease in the number of metastatic events in a B16F10 murine melanoma model, observing alterations in the expression of genes related to the cell cycle when two tumor cell lines were treated with Amblyomin-X, indicating that this protein acts selectively on tumor cells, inducing apoptotic cell death, possibly by targeting the ubiquitin–proteasome system [133].

Furthermore, the use of genomics has allowed for the identification and use of peptide sequences from scorpion and *I. ricinus* defenses (Scorpions-Ticks Defensins Ancestor, STiDA), which showed antimicrobial activities against distant pathogens related to fungal species, Gram-negative and Gram-positive bacteria, or the apicomplexan parasite *Plasmodium falciparum* [134].

As omics technologies, especially transcriptomics and proteomics, advance, other doors will open to the discovery of a wide variety of bioactive tick molecule applicable to the treatment of different diseases in animals or humans [135,136,137]. In vitro assays performed with the recombinant protein Coversin (*Ornithodoros moubata* complement inhibitor OmCl) showed that it binds to complement component 5 (C5), selectively preventing proteolytic activation of the terminal lytic pathway of the complement, making it an alternative for use in primary immunodeficiency diseases, such as inappropriate complement activation [138]. This same protein was used in a porcine model of myocardial infarction (*Sus scrofa*), obtaining a reduction in infarct size, improved ventricular function, and attenuated interleukin-1β and E-selectin by inhibiting C5 [139].

Recently, next-generation sequencing technologies have discovered different microRNA (miRNA) profiles conserved in the saliva of *O. erraticus* and *O. moubata* and different life stages of *H. longicornis* that could serve as biomarkers or genes with interesting therapeutic functions for some diseases [140,141]. These miRNAs could help delineate the regulatory signaling networks involved between pathogens and ticks or guide the development of tick vaccine candidates [122,140].

## 6. Omics to Analyze Changes in Tick Populations

Tick population changes are mainly influenced by various factors affected by climatic changes, such as host availability, habitat modifications, and the presence of human activities [142,143]. Recent advances in omics technologies have improved knowledge about the biology and genetics of ticks worldwide by being able to understand the mechanisms through which these arthropods adapt to the climatic conditions of the different areas where they are distributed (Table 1) [144,145].

Several studies have identified specific genetic markers associated with host preferences, acaricide resistances, and environmental tolerances [146,147,148,149]. For instance, genomic analysis of both *I. scapularis* and *I. ricinus* has revealed key features of their evolutionary adaptation and ecological dynamics [147,148,149,150,151]. In the case of *I. ricinus*, migratory birds enable the flow of genes through the European population and introduce new variants into the existing diversity [152].

Proteomics has served to expand information on the function of heat shock proteins (HSPs), which are used by ticks to prevent cell damage and restore normal cellular and physiological processes caused by the temperature fluctuations to which ticks are exposed [153,154,155]. Metabolomics obtained from HSPs has been able to demonstrate how the species *A. americanum*, *Dermacentor reticulatus*, *D. variabilis*, and *I. scapularis* manage energy, produce metabolites, increase different types of genes, and mitigate oxidative damage caused by temperature variations [155,156,157,158].

Likewise, the microRNAs miR-2a and miR-279, which are functionally associated with cold tolerance, have been identified in *D. silvarum*, providing insights into the mechanisms that enable such ecological adaptation [159,160,161]. In this same tick species, in the study of the genome of heat shock proteins, the genes *Dshsp70* (Hsp gene 70 of *Dermacentor silvarum*) and tubulin were identified as playing an essential role in the adaptation of *D. silvarum* to low temperatures [161,162,163,164]. Furthermore, DNA methylation is a reversible, heritable epigenetic modification some arthropods use to adapt to environmental stress [159,165]. The analysis of DNA methylation mediated by DNA methyltransferases (*Dnmts*) in *D. silvarum* and *H. longicornis* showed that the genes *DsDnmt* and *DsDnmt1* in *D. silvarum* and *HlDnmt1* and *HlDnmt* in *H. longicornis* played an important role in cold tolerance [166]. The above results contribute to understanding the survival and acclimatization of hibernating ticks.

Another important cofactor in understanding changes in tick populations is the link between the pathogens they transmit [167]. Infections with *B. burgdorferi* in *I. scapularis* affect gene expressions, influencing vector competence and immune response, demonstrating the multilayered relationship between pathogens and their host [168,169,170]. A huge number of transcripts encoding numerous distinct protein families has been identified through recent investigations of the hard tick transcriptome [74,171,172]. This demonstrated hard tick dynamic gene expression patterns in response to blood feeding, exhibiting host immune evasion, or feeding on various hosts [66,173].

On the other hand, the use of omics has allowed for the identification of specific genetic markers associated with host preferences and resistance to chemical acaricides by ticks, which has contributed to the quicker establishment of these arthropods in previously described regions [174,175,176]. For example, the use of the high-resolution quantitative polymerase chain reaction–fusion technique to identify single nucleotide polymorphisms (SNPs) of the para-sodium channel gene identified the *T2134C* mutation, which causes an amino acid change from phenylalanine to leucine at position 712, which may be associated with deltamethrin resistance by *R. microplus* [177,178].

## 7. Omics to Improve Tick Control

High-throughput discovery and characterization of tick antigens and tick-borne pathogens using vaccinomics technology combined with other analytical techniques, such as Big Data, have allowed for important recent advances in this area of research [179,180,181]. These methodologies can be used to potentially contribute to a comprehensive analysis of large datasets, which could be crucial for the selection of potential vaccine targets with high efficacy potential, resulting in the development of next-generation vaccines [179,182]. However, challenges remain, including the complexity of large omics datasets and the few immunoinformatics tools for non-model hosts, which makes challenging the analysis of the complex interactions between different tick–host–pathogen species.

Despite these obstacles, vaccinomics is allowing for the analysis and use of genome data from different tick species that have helped in the development of vaccines [179], as demonstrated by reverse vaccinology studies in *O. erraticus*, *O. moubata*, *R. microplus,* or *Rhipicephalus bursa* [183,184,185]. Through in silico analysis of tick proteins from different tissues, researchers can identify new potential antigens that can elicit strong immune responses [184,186], which need validation through in vivo vaccination assays in different conditions, such as specific host breeds and tick populations, as well as vaccine formulations. In this regard, rabbit vaccination using *O. moubata* midgut membrane proteins selected using reverse vaccinology was tested to determine their potential as vaccine targets against *O. moubata* and *O. erraticus* infestations [183,187]. Interestingly, protection was higher against *O. erraticus*, showing the potential of this strategy to support the reach of antigens against cross tick species.

One interesting use of omics technology involves the functional implication analyses of already well-characterized antigens, such as subolesin, a highly conserved tick transcription factor protein [188]. For example, transcriptomic, proteomics, and graph theory data were used in tick cells where subolesin transcripts were silenced [188]. This approach provided critical insights into the mechanisms of subolesin vaccine protection, shedding light on the gene expression regulation of specific proteins involved in intracellular transport, oxidative stress, metabolic processes and proteolysis, signal transduction, microbicidal activity, water channels, and cell stress response that can impact tick infestation efficacy.

For high-efficacy anti-tick vaccines, select conserved antigenic epitopes are crucial to minimize vaccine escape in ticks from distinct geographic areas. Through DNA sequencing and bioinformatic tools, a study analyzed the conservation of 14 tick proteins used in vaccination trials, including subolesin, from different *R. microplus* populations collected across the Americas and Pakistan [189]. The results showed significant variation in amino acid conservation across these proteins, identifying RmAQP1 (recombinant aquaporin 1 protein of *Rhipicephalus microplus*), vitellogenin receptor, serpin-1, subolesin, and the voltage-dependent anion channel as potential vaccine antigens [189].

Moreover, immunoinformatics were applied to find potential *R. microplus* and *Anaplasma marginale* protective antigens against bovine anaplasmosis [190]. This analysis identified two *A. marginale* proteins and one *R. microplus* peroxinectin, a protein involved in immunological processes that can be targeted to elicit protective immune responses to control pathogen infection. Moreover, omics analysis of the modulation of tick regulatory components in response to pathogen infections by *Anaplasma phagocytophilum* can be used for the identification of new control targets [191]. Characterizing ticks to alter their gene expression in response to pathogen infection (e.g., *A. marginale* or *A. phagocytophilum*) could help to identify vulnerabilities that could be exploited to develop new protective antigens.

## 8. Perspectives

The recent advances described in this revision highlight the potential of omics analysis as a tool to uncover gaps in different aspects of tick knowledge [192,193]. The single-cell analysis represents a new technology that can enable further high-throughput molecular profiling of tick cells. For example, using this technology, it has been possible to identify clusters of hemocyte signatures in immunity and fitness in the tick species *A. americanum* and *Ixodes dammini* [70,109]. Furthermore, the role of tick extracellular vesicles was characterized in disturbing host tissue repair via the γδ T cell–keratinocyte axis during hematophagy [194], as well as host immune responses against tick-borne diseases, such as severe fever with thrombocytopenia syndrome or Lyme disease [195,196]. In this way, single-cell technology represents a disruptive tool to understand the intricate biology of ticks, offering unprecedented insights at the cellular level that were previously inaccessible.

Likewise, multi-omics have unveiled a novel reservoir of target biomolecules that have been discovered in different tick species with immunomodulation, antimicrobial, anticoagulant, anti-inflammatory, and antitumor functions in different tumor cell lines. These molecular biology technologies have opened new lines of research at the pharmacological level, analyzing ticks not only as ectoparasites and vectors of pathogens but as an excellent source of new molecules with a variety of functions and therapeutic properties. However, despite the fact that a variety of possible therapies using biomolecules from ticks have been identified, no studies have been continued that could lead to the approval of any type of new drug by federal health agencies, such as the Food and Drug Administration (FDA) in the United States [137].

It is expected that in the coming years, other types of technologies, such as spatial omics and holo-omics, can be used, combined with advances in single-cell omics that allow for a better understanding of the interactions between different cells and molecular distributions or their relationship with the tick microbiota [197,198]. Finally, the development of faster, high-performance, and lower-cost sequencing technologies can be integrated with already advanced artificial intelligence tools for data analysis, opening new avenues for research on this critical pathogen vector in both animal and human health.

## Figures and Tables

**Table 1:** Application of omics to improve tick research.

Area	Omics	Technology ^1^	Tick	Highlights	Ref.
Feeding and Digestion					
	Proteomic	LC-MS/MS	*Amblyomma americanum*	Profile of tick saliva proteins during different phases of the tick feeding process	[18]
	Transcriptomics	RNA-seq	*Ixodes scapularis*	Morphological changes in tick midgut are accomplished through transcriptional changes	[19]
	Transcriptomics	RNA-seq	*Ornithodoros hermsi*	Unfed soft ticks intensify the transcription of genes related to blood feeding/digestion prior to the blood meal	[20]
	Transcriptomics	sc-RNA-seq	*A. americanum*	Hemocyte heterogeneity in blood-feeding tick and changes in *Ehrlichia*-infected hemocytes	[21]
Reproduction and Embryology					
	Proteomic	LC-MS/MS	*R. microplus*	Protein profile during ovary maturation	[22,23]
	Transcriptomic	RNA-seq	*Ixodes ricinus*	Importance of ovaries as molting regulators	[24]
	Transcriptomic	RNA-seq	*R. turanicus*	Gene expression profiles at different stages in embryonic development	[15]
Neural and Endocrine Regulation					
	Transcriptomic		*I. ricinus*	Evolution of the cys-loop ion-ligand channel family	[25]
Tick Control					
	Transcriptomic and proteomic	RNA-seq and LC-MS	*R. microplus*	Transcripts and protein profile of salivary glands are affected by developmental stage and the source of blood	[26]
	Transcriptomic	RNA-seq	*Dermacentor nuttalli*	Transcriptome composition shows variation through the life cycle	[27]
Bacterial Microbiota					
	Metagenomic	WGS	*Rhipicephalus sanguineus* s.l. and *R. turanicus*	Implications of tick microbiota in rickettsial diseases	[28]
	Transcriptomics	RNA-seq	*Amblyomma maculatum*	*Rickettsia parkeri* infects hemocytes to modify tick cellular immune response	[29]
Changes in Tick Populations					
	Genomics	NGS	*I. ricinus*, *Ixodes persulcatus*, *Ixodes pacificus,* and *Ixodes hexagonus*	Improve the understand of gene evolution in tick biology	[30]

^1^ Liquid chromatography: LC. Mass spectrometry: MS. RNA sequencing: RNA-seq. Longitudinal single-cell RNA-seq: sc-RNA-seq. Next-generation sequencing: NGS. Whole Genome Sequencing: WGS.

## Data Availability

No new data were created or analyzed in this study. Data sharing is not applicable to this article.

## Supplementary Materials

The following supporting information can be downloaded at https://www.mdpi.com/article/10.3390/microorganisms13040795/s1, Figure S1: PRISMA flowchart used to collect data and select reports in this study.
