# Supplementary figures and images for: Multi-Omics Technologies Applied to Improve Tick Research
Source: Microorganisms. 2025 Mar 31;13(4):795. doi: 10.3390/microorganisms13040795 (PMC12029647; doi:10.3390/microorganisms13040795)

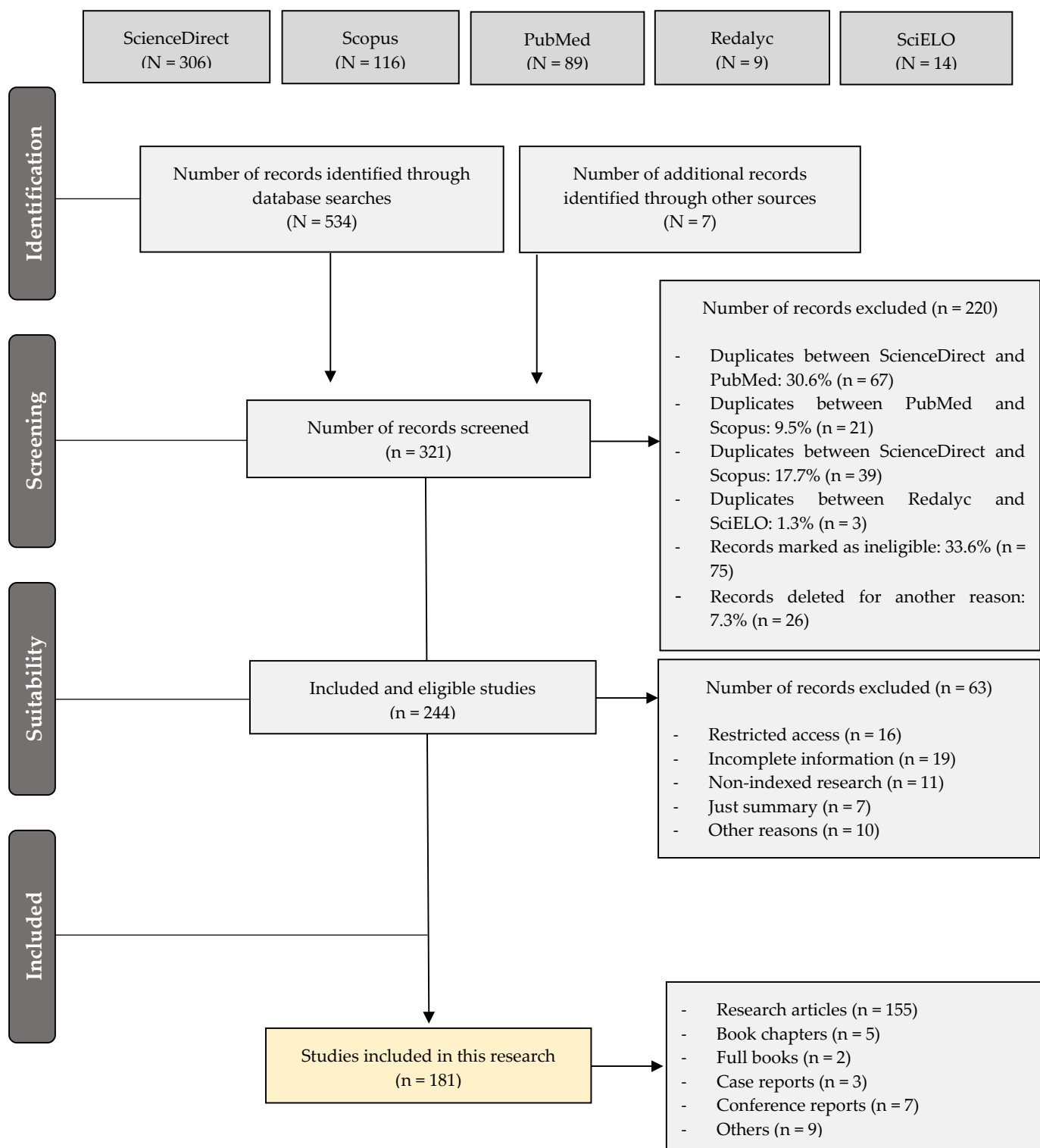

**Figure S1.** PRISMA flowchart was used to collect data and select reports in this study.

Supplement: Supplementary file 1 [file microorganisms-13-00795-s001.zip › microorganisms-3486257-supplementary.pdf]
